# Climatic pluriverses: community contributions to socio-ecological resilience from intercultural territories within climate-extractive entanglements in south-central Chile

**DOI:** 10.3389/fpubh.2026.1841434

**Published:** 2026-07-30

**Authors:** Bárbara Jerez-Henríquez, Pablo Mansilla-Quiñones, Janny Figueroa-Ayala, Gerson Castillo-Queupul, Andrea Monica D. Ortiz

**Affiliations:** 1 Centro de Estudios del Desarrollo Regional y Políticas Públicas - CEDER, Universidad de Los Lagos, Osorno, Chile; 2 Instituto de Geografía, Pontificia Universidad Católica de Valparaíso, Valparaiso, Chile; 3 ONG We Kimün, Santa Bárbara, Biobío, Chile; 4 Escuela de Trabajo Social, Universidad del Bio Bio, Concepción, Chile; 5 Departamento de Geografía, Facultad de Arquitectura, Urbanismo y Geografía, Universidad de Concepción, Concepción, Chile

**Keywords:** climate change, climatic pluriverses, climatic-extractive entanglements, local communities, socioecological resilience

## Abstract

**Introduction:**

The climate change crisis intensifies territorial inequalities and gives rise to diverse community responses oriented toward environmental justice, which we term climatic pluriverses. These are initiatives grounded in local knowledges, practices, and ontologies that propose alternatives to universalizing global technopolitical approaches. Within this framework, the Biobío region (Chile), a territory historically shaped by territorial conflicts between the Mapuche people and the Chilean State, presents high climate vulnerability and marked socio-environmental degradation produced by extractive dynamics. We define this intersection of threats as climate-extractive entanglements, whose complexity has exacerbated structural inequalities and strengthened community territorial defenses.

**Methods:**

This article analyzes pluriversal experiences through an interdisciplinary approach based on a georeferenced inventory and ethnographic fieldwork grounded in geonarratives across Andean foothill, dryland, and coastal territories.

**Results and discussion:**

The results show that these experiences express forms of socioecological resilience based on deep territorial rootedness and ecologically interdependent ties with ecosystems, deploying diverse strategies in response to climate–extractive entanglements. Despite the limitations on collective action, climate pluriverses emerge as alternatives co-produced with nature and with affective rootedness in place that make it possible to rethink community resilience from its socioecological, relational, and decolonial dimensions, contributing new perspectives to academic debate and to the design of climate strategies in interculturally diverse and ecologically degraded territories.

## 1 Introduction

### 1.1 Environmental crises and climatic pluriverses

Climate change constitutes an essential dimension of the multiple socio-environmental crises and represents one of the most urgent challenges of our time (1). The impacts of these crises are profoundly unequal because of the asymmetrical distribution of greenhouse gas emissions between the Global North and the Global South. This inequality has particularly affected communities that have historically been exposed to greater social and environmental vulnerability (2). These same communities also endure persistent systems of structural oppression, racism, and colonialism (3). In this sense, the coloniality of climate change reproduces power structures over Indigenous peoples, peasant communities, and Afro-descendant communities, who have been systematically marginalized by the development project of modernity (4).

At the same time, the hegemonic governmental solutions designed to respond to the climate crisis often impose forms of epistemic violence and new forms of oppression (5). By establishing a universalizing scientific and technical narrative to address climate change, these policies exclude local groups as valid interlocutors and disregard their situated practices and knowledges (6). This hegemonic account offers solutions that continue to operate within the frameworks of a new capitalist colonial geopolitics (7), depoliticizing ecological debate and avoiding any questioning of the forms of nature destruction that gave rise to the current crisis (8, 9).

However, in the face of this neoliberal climate governance and apocalyptic future narratives, community practices and perspectives emerge that propose a cosmopolitical construction of our relations in times of global social, ecological, and biophysical transformations: the concept of the pluriverse. Based on the formulations of Arturo Escobar (10) and Kothari et al. (11), the pluriverse highlights multiple ways of inhabiting, knowing, and transforming the world that emerge from local scenarios of the contemporary environmental crisis. These experiences hold profound transformative potential, grounded in interaction and interdependence with diverse human and more than human forms of life (12).

The pluriverse emerges as an articulating concept of ontologies and political praxes that shape community alternatives based on *buen vivir*, relationality, and autonomy, which exceed conventional institutionalized responses to climate crises and project alternative horizons (11) that are inscribed within a broader civilizational crisis (10). Far from a romantic perspective, climate pluriverses recognize the tensions and limits of the concept itself, underscoring the need to build emancipatory projects that contest modern, colonial, and capitalist power structures (13).

This article aims to explore the notion of pluriverses as a situated and contextualized analytical and political-epistemic concept in relation to the climate crisis and multiple extractivisms in the territory, seeking to highlight the role of communities and nature in processes of resilience and resistance in the face of these dynamics. By situating this concept in the territory, the article seeks to unravel how, under the hegemony of technocratic solutions, territorial practices and knowledges configure a socioecological resilience capable of integrating local responses to complex climate-extractive entanglements.

Through the analysis of experiences and perspectives, this study seeks to answer the following question: How do the practices and local knowledges of climate pluriverses configure a field of socioecological resilience capable of integrating effective responses to the entanglements between climate change and multiple extractivisms?

To this end, the study focuses on the Biobío region, located in south-central Chile, which for centuries has been an active frontier of dispossession, territorial diplomacy, and forced coexistence between the Mapuche people, the Spanish Crown, and the Chilean State. At present, this complexity persists in power asymmetries and in high levels of conflict surrounding multiple forms of forestry and energy extractivism, within a framework of constant socio-environmental disasters exacerbated by the climate crisis (14).

In this region, the long history of territorial disputes has operated as a structuring condition (15). There, longstanding memories of dispossession converge with transformed ecologies and multiple experiences of care that persist creatively. These practices of defense and care generate resilience and reimagine the relations between society, nature, and power. Owing to its geography, the region brings together the lived realities of communities in the Andean foothills, dryland areas, and coastal zones.

**Figure 1:**
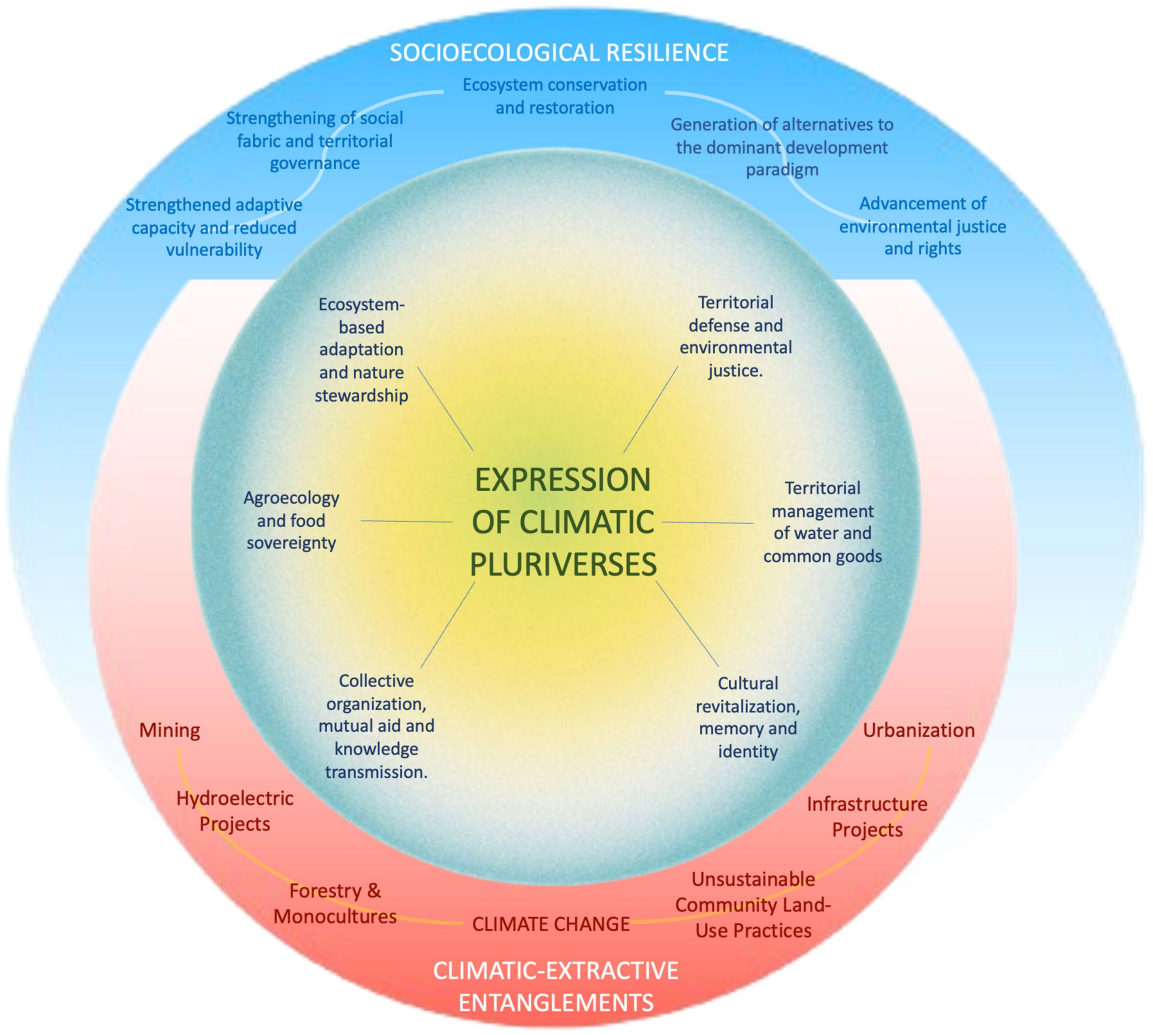
Expressions of climate pluriverses in the face of clmatic-extractive entanglements that generate ecological resilience.

The territory is marked by the impacts of a persistent megadrought and a succession of high-intensity mega-fires (16). Specifically, the events of 2017, 2023, and January 2026 have been devastating for peasant communities, Mapuche communities, and sectors of the urban-forest interface. Recent fires in Penco, Tomé, and the rural areas of Concepción reveal the synergy between the climate crisis and an intensified forestry-energy extractive model (17). This scenario increases socio-environmental vulnerability in a territory where the expansion of heterogeneous extractive models converges with the absence of resilient territorial planning (14). In this context, community experiences of care emerge from social fabrics shaped by intercultural encounters and by the historical accumulation of responses to these structural convergences (Figure 1).

### 1.2 Socioecological resilience and pluriverse

Socioecological resilience it is defined as the capacity of a socioecological system to absorb disturbances, reorganize, and maintain essentially the same function, structure, and identity (18). At the same time, authors such as Montalba et al. (19) define resilience from a community-based perspective in rural territories as *“the capacity of a farming system to maintain its core functions, identity, and adaptive practices under disturbance, rather than as the mere inverse of vulnerability.”*

Within the framework of political ecology perspectives (20), this concept has evolved from a view focused solely on persistence toward one centered on transformative capacity. In this sense, socioecological resilience refers to the capacity of social actors to learn, innovate, and actively transform the system when prevailing conditions become unsustainable or unjust (21). The resilience capacities that communities generate in contexts of climate crisis are key to the “*propensity of a system to retain its organizational structure and productivity after a disturbance*” ((22):18) and to the strengthening of ecological heterogeneity (23).

From this perspective, resilience is not a strictly biophysical phenomenon, but is directly related to the social resilience of the communities that inhabit the territory. It is expressed in the interconnections among traditional governance systems, traditional knowledges, and ancestral practices of organization and social reciprocity (22). Thus, pluriversal experiences constitute a platform of socioecological resilience that emerges from ecopolitical discussions within agroecology, addressing agricultural, ecological, cultural, social, and economic dimensions (22–24). In this approach, the biophysical and sociocultural dimensions have a relational and ecodependent character, in which affectivity toward place constructs situated resilience (25, 26).

Critical political ecology studies have addressed resilience within the broader power structures that shape the environmental disturbances affecting communities. For example, from a critical and decolonial perspective, Santiago-Vera et al. (27) argue that peasant resilience promotes alternatives to capitalist forms of labor organization and land use through struggles for autonomy. In these contexts, resilience is generated through agroecological practices and forms of resistance to hegemonic development models. Along similar lines, Holt-Giménez et al. (28) analyze peasants’ resilience in the face of natural disasters, arguing that fostering resilience requires more than the implementation of biophysical adaptation strategies for crops. Rather, it demands addressing the structural causes of vulnerability, highlighting the need to confront long-standing issues of agrarian justice, food sovereignty, and equitable access to land.

Similarly, from a feminist political ecology perspective, Garcia et al. (2) examine how social hierarchies shape which forms of knowledge are legitimized and which voices are marginalized in resilience processes. Accordingly, resilience should be understood as a sociopolitical process of continuous negotiation among diverse actors, requiring attention to three key questions: (i) who holds political authority over knowledge production in resilience processes?; (ii) from a politics-of-scale perspective, who defines the appropriate level of intervention?; and (iii) how are subjects classified as vulnerable or resilient?

Likewise, Sou (29) analyzes the case of Puerto Rico following the devastation caused by Hurricane Maria, where colonial relations of dependence on the United States were reaffirmed. According to the author, this case underscores the need to reconceptualize resilience as a form of political resistance against the structures of contemporary colonialism.

From a different yet equally critical perspective, Arianna Tozzi (30) approaches resilience through a pluriversal lens, drawing on political ontology to question the concept of Social-Ecological Systems as a functionalist and universalist narrative that reinforces a one-world ontology. In this regard, she asks: *What forms of resilience do we allow to exist?* Tozzi proposes a pluriversal understanding of resilience that: (i) recognizes multiple ways of being, knowing, and responding to crises; (ii) contributes to the decolonization of the Western resilience tradition; and (iii) creates space for marginalized resilience practices and voices.

In our own approach, when we speak of ‘climatic pluriverses’, we are referring to the coexistence of multiple territorial rationalities (25), relational ontologies and situated ways of understanding, inhabiting and acting in scenarios of climate crisis, where the emphasis lies on the diversity of possible socio-ecological worlds that emerge from this. For its part, socio-ecological resilience—drawing on the contributions of political agroecology—refers more specifically to those territorial capacities for adaptation, regeneration and community reorganization in the face of climatic and environmental disturbances, with a particular focus on agroecological practices, local autonomy, biodiversity and strategies for the reproduction of multi-species life.

In this way, climatic pluriverses constitute an epistemic, ontological and political framework that recognizes the legitimacy of multiple forms of existence and knowledge arising from community responses to the climate crisis; socio-ecological resilience represents a more practical and territorial dimension in which these ontologies and forms of knowledge materialise in concrete responses of a biocultural and politically relational nature.

The scientific literature documents a broad diversity of research on pluriverses that examines alternative experiences of relating to nature and development. However, it also reveals a significant tendency for research to concentrate in Latin America, together with fragmentation across cases and limited systematization of community resilience experiences promoted by Indigenous peoples, peasants, and urban popular sectors. Among these, the work of Schingmann et al. (31) highlights adaptive capacity and territorial rootedness, as does the proposal of Kothari et al. (11), who conceptualize these alternatives through the notion of the pluriverse. These experiences configure practices oriented toward just transitions (32, 33) and territorial responses to socio-environmental conflicts, promoting the collectivization of transformative experiences (34).

In Latin America, the work of Sandoval (35) stands out, defining pluriverses from the perspective of socio-natural disasters as fields of resistance grounded in the very places of community “*ser-estar*,” of permanence and being. Also in Brazil, the work of Almeida et al. (32) analyzes the ways in which “ecovillage” projects of territorial ordering and community life, grounded in permaculture, agroecology, and bioconstruction, represent experiences of community resilience in the face of climate change. The work of Quintero-Weir et al. (5), with the Wayuu Indigenous People, analyzes the pluriversal perspectives that their inhabitants articulate in response to the rupture of the community calendar produced by climate change. Also in Chile, the work of Llancamán (36) stands out, proposing the notion of Indigenous pluriverses in relation to cosmovisions of the commons. In relation to the climate change crisis, the works of Blanco (37) are particularly relevant, proposing “pluriverses of conservation” based on care among entities in a more-than-human world. Likewise, Jerez et al. (38, 39) propose the concept of (neo) peasant pluriverses in relation to the resilience provided by traditional agroecological knowledges in the face of the climate crisis in vulnerable rural territories of south-central Chile.

Finally, this approach contributes to strengthening community response capacity to buffer the effects of environmental variability (40). These experiences are usually located in territories affected by processes of socio-environmental degradation, a concept under which we group the accumulation of multiple dispossessions, local overexploitation, and disasters that intensify local climate vulnerability.

### 1.3 Climate change and climate-extractive entanglements

Local climate vulnerability transcends its biophysical dimension to configure a field of new representations, social practices, and collective subjectivities. In this space, not only are responsibilities for the crisis disputed, but meanings and future horizons are also contested in scenarios of high uncertainty (41). Climate debate intertwines divergent visions of sustainability and governance that reveal power asymmetries linked to structural inequalities, confronting hegemonic development models with alternative ways of inhabiting the territory (42, 43).

From critical environmental sociology, climate change is inscribed within the civilizational crisis of contemporary capitalism (44), linked to the dominant systems of knowledge that have sustained industrial and extractive expansion since the Anthropocene (43, 45). These dynamics have intensified ecosystem degradation, producing new configurations of socioecological suffering that exceed the regenerative capacity of the natural cycles of the Earth (46). Thus, the phenomenon becomes a crisis of hegemonic forms of society–nature relations, opening fundamental questions about the possible bifurcations of the future.

Political ecology, in turn, analyzes climate change through the dynamics of global accumulation and coloniality. This discipline examines how unequal geographies of vulnerability and risk are configured, disproportionately affecting Indigenous communities, peasant communities, and popular sectors (4, 47, 48). Within this approach, the technocratic frameworks of global climate governance are problematized, denouncing how they tend to render territorial conflicts and local knowledges invisible.

From these perspectives, climate change is a field of tensions and materialities in which climate and epistemological injustices are reproduced (49), confronting projects of society embedded in diverse pluriverses and transition horizons.

In territories marked by high levels of environmental degradation, such as the Biobío region, these horizons emerge from the convergence and feedback of multiple socio-environmental threats, whose articulation we conceptualize as climate-extractive entanglements. These entanglements constitute a relational field where the climate crisis, the intensification of socio-natural disasters, and the superposition of diverse extractivisms (forestry, mining, or energy), together with the expansion of invasive species and the overexploitation of commons, coexist and generate synergies (50).

These entanglements form a network of interdependencies that produces complex scenarios of socio-environmental crisis (51), characterized by territorial dispossession (52) and the violation of local ways of life. As they become intertwined with socioeconomic inequalities and high poverty rates, these dynamics deepen conditions of risk for the communities that inhabit these territories (53). Within this framework, climate-extractive entanglements reveal the structural and cumulative dimensions of the contemporary socio-environmental crisis, as well as the urgency of imagining and strengthening socioecological transitions that are territorially situated and socially just.

### 1.4 Climatic pluriverse experiences and study approach

Epistemological debates on multiple crises and the diverse and divergent ways of addressing them configure a fertile field of alternatives for the construction of socioecologically resilient and sustainable futures. Within these debates, the dominant rationalities of development are disputed, creating opportunities to reconfigure the relations between society, nature, and economy in ways that sustain life (54). Under this scenario, climate-extractive entanglements act as the point of origin of diverse resilience practices and new “re-existences” in the face of crisis (10, 78), which we call pluriversal experiences. Within them, biocultural synergies are produced between local knowledges accumulated in disputed territories and the dialogue of knowledges (54), mobilizing territorialized knowledge to find responses to the crisis and build processes of socio-environmental recovery (55).

This accumulation of community responses that emerges from the adversity of these entanglements constitutes what we have termed climate pluriverses. As indicated above, the idea of the pluriverse arises in response to the homogenizing conceptions of Western and colonial thought, based on a “universe” knowable only through modern science and a Eurocentric worldview (11, 56). As a concept, the pluriverse makes visible the infinity of alternatives self-defined by communities and social movements to confront a climate crisis that often intensifies previous trajectories of environmental injustice (57).

Climatic pluriverses therefore imply a heterogeneous field of projects of a cosmopolitical character anchored in practices and socio-territorial fabrics; they are configured as active responses to the deepening of socioecological crises. Central to territorial responses are relational ontologies and rooted sociospatial practices, from which collective assemblages are deployed and co-produced through the specific bond of care that communities weave with their places.

In this sense, climatic pluriverses exceed the universalizing technopolitical rationalities that define and implement global decisions on climate change. Far from constituting idealized experiences (34), they comprise a continuum of practices with different degrees of transformative potential. These emerge from the materialities and subjectivities that derive from everyday life within climate-extractive entanglements and, in doing so, open concrete horizons for the construction of more just and care-oriented socioecological futures (11, 58).

However, despite the potential of these experiences, a gap persists in understanding how climatic pluriverses manage to sustain themselves and scale up in the face of long-term extractive pressures, particularly in the Southern Cone.

## 2 Methodology

The study adopted a qualitative and spatial methodology within decolonial and interdisciplinary perspectives at the intersection of the social sciences, the humanities, and geography (12, 59, 60). To this end, two complementary research techniques were employed. First, an inventory was developed to map emerging socioecological practices in the Biobío region.

### 2.1 Inventory and mapping of community climatic experiences

These were identified as community climate experiences from 2024 to the end of 2025 under three selection criteria:

- Community leadership in places threatened by climate-extractive entanglements.
- Territorial care actions grounded in local rootedness.
- The generation of situated responses to the climate and socio-environmental crisis.

The initiatives were selected by first identifying those known to the research team using the snowball sampling method; the team then asked a group of socio-environmental organizations if they were aware of any other initiatives; and finally, a search was conducted on social media in each municipality within the region, prioritising those that met the criteria for inclusion in the inventory.

These experiences were then georeferenced using ArcGIS Pro software. The resulting spatial analysis made it possible to identify and understand the distribution of the experiences in relation to territorial characteristics and extractive pressures. The information generated by the inventory and georeferencing contributed to a multiscalar reading of the pluriversal experiences and guided the selection of cases for the second ethnographic component of the study.

### 2.2 Fieldwork and geonarratives

To select the initiatives to be interviewed, a purposive sample of 12 community-led socio-environmental conservation initiatives located in the Biobío River basin, in the Biobío Region (Chile), was considered between 2024 and 2025. For each initiative, guided visits were conducted with the organizations at the environmental care sites, and in a second stage, face-to-face interviews were held with a leader or active participant from each organization, all of whom are local residents of the territories where the community initiatives are based. According to Quintero-Weir et al. (5), geonarratives comprise situated accounts through which social actors produce and interpret the meaning of their territories from place, articulating lived experience with historical memory, local knowledges, and socioecological relations.

Unlike abstract narratives, geonarratives are deeply rooted and situated in territorial contexts. From geography, territory is understood not as a mere physical support, but as a relational production constituted by practices, discourses, and affects (61). Thus, geonarratives constitute a key methodological tool for understanding how communities interpret territorial transformations, develop resilience strategies, and project alternatives in contexts of high vulnerability.

The prioritisation of case studies was determined by taking into account theoretical and territorial criteria aimed at ensuring socio-ecological and territorial diversity. The selection criteria considered in choosing the community initiatives were as follows:

1) Self-managed initiatives led by community groups in dryland, coastal and foothill areas threatened by climate-extractive dynamics in the Biobío River basin.
2) Territorial representativeness of ecosystems and socio-territorial configurations in the Biobío River basin: Initiatives in dryland, foothill and coastal areas.
3) Territorial representativeness of ecosystems and socio-territorial configurations in the Biobío River basin: Initiatives in dryland, foothill and coastal areas.
4) Initiatives that generate locally-based responses to the climate and socio-environmental crisis in their territory.
5) Willingness and interest of each community group to participate in this study.
6) Representativeness of the diversity of practices and strategies for socio-environmental care activities.

In the case of community initiatives located in dryland and foothill areas, those interviewed were residents of rural areas, mostly of peasant origin, migrants from large cities who had settled in the area, and, in a minority of cases, members of the Pehuenche indigenous people in the foothills. In contrast, in the coastal areas, the participants were non-indigenous residents of semi-urban areas.

The selection of the initiatives studied was carried out in three stages: (1) Based on the previously compiled inventory of community initiatives in the Biobío river basin, the research team reviewed documentary records of the initiatives, local networks and prior knowledge of each organization’s activities. (2) In a second stage, the initiatives were discussed and prioritised collectively by the research team, ensuring compliance with the previously outlined selection criteria. (3) Finally, the organizations were contacted via telephone calls and social media to invite them to participate in the research, explaining its objectives, the nature of the fieldwork process, and its ethical considerations.

The interviews were recorded and transcribed, and lasted between one hour and one and a half hours. Prior to the interview, participants were informed that they would be asked to sign a written informed consent form, which guarantees the confidentiality of their accounts, their voluntary participation and the right to withdraw if they felt uncomfortable with any question, as well as the use of the information strictly for scientific purposes and its storage on computers in virtual folders accessible only to the research team. The analysed information is recorded in documents without naming individuals, identifying each interviewee by a number and the type of territory (drylands, foothills, coast). This informed consent was part of the ethical review carried out on the publicly funded research project (National Agency for Research and Development, Chile) Anillo ATE230072 by the ethics committee of the Pontificia Universidad Católica of Valparaíso, Chile.

In the case of members of indigenous communities, the research was conducted in accordance with ethical principles of respect, voluntary participation and cultural sensitivity. Prior to each interview, a relaxed and informal conversation took place with the participants, who had a full understanding of the Spanish language and chose to communicate in that language. The aim of the study was then explained to them and their written informed consent was sought; this was read out and discussed. Interactions were conducted using accessible, respectful language adapted to the participants’ pace and communication styles, as well as to their worldviews regarding the territory and its elements.

Given the qualitative and purposeful nature of the sample, the results of this study do not seek to provide a statistical representation of all territorial experiences within the Biobío River basin, but rather to gain a context-specific understanding of the community’s perspective on the socio-environmental transformations of the territory, the specific characteristics of community horizons and their experiences regarding socio-ecological resilience in contexts of high environmental and social vulnerability in Chile.

#### 2.2.1 Data analysis

The interviews were transcribed in full and analysed manually using a reflexive thematic coding strategy, but which remains open to the inclusion of new categories that may emerge during the research process (62, 63). This process was organized around the following categories of analysis, developed from the theoretical framework, the research question and the problem statement, and which also formed the basis of the interview guide used: (1) Socio-environmental characteristics of the territories; (2) Socio-ecological interrelationships between communities and territory; (3) Transformations associated with climate change; (4) Impacts arising from extractive activities and overexploitation; (5) Community experiences and socio-ecological resilience; (6) Future projections for the territories and their communities.

The information was subsequently operationalised by identifying the most recurrent patterns across the board and for each territory (drylands, coast, foothills) based on the communities’ narratives. Within the thematic analysis of each category, emerging classifications (62) also began to emerge, which we identified and included; subsequently, the most representative accounts from all classifications were grouped together. The generation and analysis of the information continued until sufficient interpretative density and analytical recurrence were achieved regarding the socio-ecological dynamics identified by territorial actors in each category and in each territory.

### 2.3 Study territory

The Biobío region presents high exposure to climate risks associated with biodiversity loss and with projections that indicate increased drought and reduced precipitation, together with stronger winds and storm surges, and the reduction of coastal strips (64, 65). Likewise, the region presents high exposure to socio-natural disasters, as it has been affected by earthquakes, tsunamis, storm surges, and fires (16, 64, 65).

The region also faces a high recurrence of socio-natural disasters such as earthquakes, tsunamis, and fires (16, 64). Particularly notable is the high incidence of summer fires, which between 2022 and 2023 affected nearly all rural municipalities in the region. More recently, in the summer of 2026, new forest fires of unprecedented magnitude spread across a large part of coastal municipalities such as Penco and Tomé, causing thousands of people to lose their homes and leading to the loss of important ecological reserves (66).

The socio-environmental impacts associated with climate change and the dominant development models in the region are concentrated in territories marked by high social, environmental, and historical vulnerability, which together produce scenarios of climate sacrifice. These territories contain diverse peasant and Indigenous, marine-coastal, urban popular, and forestry-mining identity and productive configurations that have been permanently strained by extractive projects and exogenous territorial planning (67, 68).

In this context, the historical expansion of pine and eucalyptus forest monoculture plantations in recent decades has not only profoundly reconfigured local landscapes and economies, but has also contributed to intensifying the water crisis, biodiversity loss, and the increased risk of forest fires (16). These dynamics are inscribed in a region marked by medium and high levels of poverty (69), which reveals the interdependence between social inequality, environmental degradation, and climate vulnerability.

Closely linked to the above, the Biobío region exhibits a high intensity and frequency of socio-environmental conflict and a dense organizational fabric that actively disputes the meanings of territory, nature, and socioecological transitions (70). In this context, diverse local organizations articulate processes of territorial defense, ecological restoration, and the generation of alternatives to the extractive model, configuring community climatic experiences in action, where multiple ontologies, knowledge, and situated practices converge.

Thus, this territory emerges as a strategic socio-environmental laboratory for understanding the configuration and local answers of pluriverses in scenarios marked by the historical superposition of extractivisms and growing climate risks under conditions of social inequality.

## 3 Results

### 3.1 An inventory-based analysis of community climatic experiences in the Biobío region

A key factor for understanding the proliferation of community climatic initiatives in the Biobío region is the expansion of pine and eucalyptus monoculture plantations, which represents one of the main drivers of land use change and socio-environmental conflict in Chile, especially in the south-central part of the country (71). Added to this is the emblematic conflict in the Andean foothill sectors of the region associated with the construction of 8 hydroelectric dams (Abanico, Antuco, El Toro in the Laja River; in the Biobío River there are Ralco, Quilleco, Angostura, Pangue, and Rucalhue under construction), whose expansion is still underway and has generated ongoing tensions with Pehuenche communities in the territory (79). These events marked a turning point of such magnitude that they contributed to the creation of the current Ministry of the Environment in 2012, and even in some reforms to public policy concerning Indigenous peoples (72).

The region has a significant critical socio-environmental mass that emerges in scenarios of intense territorial conflict and collective activism expressed in community climatic experiences. Within these, the care of wetlands and rivers, environmental education initiatives, environmental restoration practices, agroecological experiences, environmental defense, and seed care are especially prominent. These are primarily experiences driven by local actors—especially women and young people—who articulate networks, mobilize diverse knowledges, and promote strategies of community environmental defense. In general, local collectives, environmental groups, and territorial coordinating bodies predominate, and to a lesser extent foundations and NGOs that emerge in response to environmental threats.

### 3.2 Socio-environmental characteristics of the territories

**Figure 2:**
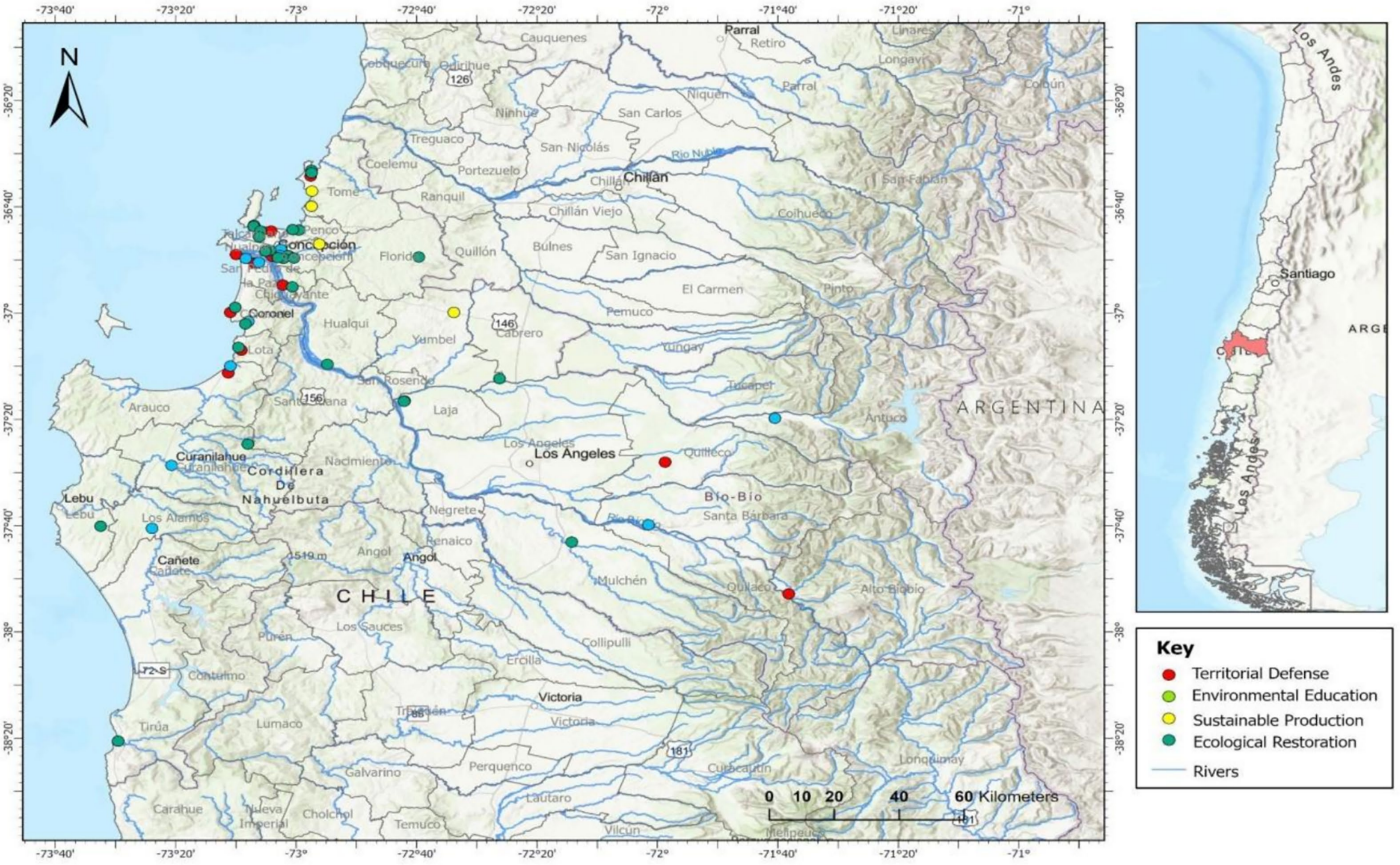
Map of the Biobío region, with points marking the presence of organizations engaged in territorial defense, environmental education, sustainable production, and ecological restoration. Source: prepared by the authors.

The pluriversal experiences analyzed are located in coastal, dryland, and Andean foothill territories of the Biobío region (Figure 2), where high biocultural richness intersects with significant socioecological degradation and vulnerability. This vulnerability is associated mainly with the expansion of large-scale extractive activities such as forest monocultures, hydroelectric projects, and real estate expansion, which intensify ecosystem degradation and fragmentation.

In the Andean foothill territories of the region, there is high biodiversity (73), sustained by glacial systems and a dense fluvial network that supports highly complex native forests. However, the geonarrative testimonies indicate that local ecosystems face strong environmental pressures stemming from eucalyptus and pine monoculture plantations, and from hydroelectric projects that dam mountain rivers.

*“…It is a mountain range where a lot of water originates, a lot of springs. Those springs feed the main rivers… Pangue, the Bío-Bío, and the Queuco”* (Interview representative organization Malen Leubu, Alto Biobío).

In the dryland territories of the central valleys of the region, water availability is limited, ecosystems are highly fragmented, and the economic matrix is dominated by medium- and high-impact agricultural and forestry activities, which increases susceptibility to the impacts of environmental degradation and the climate crisis. There is also a coexistence of native forests and forest monocultures, together with a growing presence of large wind farms and hydroelectric plants (74).

The community experiences interpret these processes as a growing tension between the expansion of productive development models and the conservation of native ecosystems across all the territories studied in the Biobío region, where biodiversity remnants are identified as increasingly scarce:

*“…well, there is a lot of countryside, but it is countryside that is also being heavily intervened by forestry, and that takes away all its ecological strength, since it is being devastated by the forestry companies, socially and ecologically…. several forests are practically being destroyed… There are many forestry properties around what is La Orilla…”* (Interview environmental activist, La Orilla, Quilaco).

In coastal territories, urban and semi-urban areas predominate, where wetlands sustain high biodiversity in the surrounding areas of Greater Concepción. The proximity between human settlements and sensitive ecosystems generates intense interactions and conflicts over territorial use. Processes such as urban, industrial, and forestry expansion—together with wetland filling—have increased pressure on these systems, exacerbating flooding, habitability problems, and urban fragmentation. This highlights their key role in water regulation and in the mitigation of socio-environmental risks:

*“At least in my sector, the wetland is rich in biodiversity, and at the same time people in my neighborhood became aware that we depend on the wetland so we do not get flooded… but as the city grew we ended up kind of boxed in… then in 2006 they blocked that outlet. And that caused flooding…”* (Interview leader Chimalfe wetland-Hualpén).

### 3.3 Socioecological interrelations between climatic community experiences and territory

The analysis of this community experiences shows that the bond between community and nature rests on a strong territorial rootedness, built both by local inhabitants born in these places and by migrant actors from other cities and regions who have forged forms of attachment in the territory. In both cases, the territory is configured as a material and symbolic support of everyday life with nature, charged with meanings, affects, and social relations that articulate collective identities (75).

This rootedness is expressed in historical belonging to place—characteristic of those who have inhabited the territory for generations—but also in forms of attachment and rootedness constructed by actors who, through migratory processes, decided to establish their lives in these spaces. In both cases, the territory becomes a material support for everyday life charged with meanings, affects, and social relations that articulate the collective identity of these experiences, in which socio-environmental care is an extension of those relations. This fabric linking territory, community, and lived experience as “landscape and collective memories” is expressed in the following account:

*“Well, I have never really left… sometimes I go away on a short trip, but I feel I love this place, I have always loved it and that is why I am here… all your friends are here, you have known them since you were little… on this land we are all family despite our differences many times… everyone here knows me…”* (Interviewee, group in defense of the Laja River and the Environment, La Aguada, Yumbel).

Among communities belonging to the Pehuenche Indigenous People, there emerges an ancestral relation of reciprocity and interdependence in which people and nature are one, and this was the worldview that predominated in the Pehuenche world of the Alto Biobío in the past:

*“We lived depending on one another… In protecting the rivers, in protecting our forests, in the fact that we practiced transhumance… When you say with nature, we were one, you are speaking in the past.”* (Interview Pehuenche representative Ulkantún, Alto Biobío).

Many actors in these pluriversal experiences migrated to these territories at different stages of life, building a bond with nature not from historical belonging, but through processes of learning, adaptation, and everyday coexistence. In this way, the territory is resignified as a personal and collective space, where the relation with nature is forged through interaction with local cycles and practices, oriented both toward subsistence and socio-environmental care, generating an integral connection with the ecosystem.

*“My relationship with the environment since I came here has been a life relationship, really, one of sustaining myself… Learning to move and live according to the cycles it has… my relationship with the environment is completely reciprocal and constant, because I like to grow things… I grow my own food.”* (Interview environmental activist, La Orilla, Quilaco).

In the case of urban wetlands on the coast, both the emotional bond with the territory and the importance of enjoying it and caring for it are central, since it is considered a common good of the community and one of the few spaces that still holds biodiversity. For that reason, it must be cared for and defended from the threats surrounding it:

*“…on the one hand, as people from the neighborhood, we really enjoy our leisure time in the wetland… that led to our involvement in defending the wetland… there is also an impact on the surrounding communities, because it is their leisure space. A place to go spend the afternoon, walk the dog.”* (Interview leader Paicaví wetland, Concepción).

Rootedness in place under conditions of environmental threat contributed to strengthening the socio-natural affective bonds that many of these actors have with place and with its species, and led them to undertake processes of care and environmental defense that have also become part of everyday life:

*“It is my child… (the wetland)… it is more than anything a commitment… because… when I came to live in this neighborhood, it was the last house, I had the wetland right beside me and almost directly in front of me. And of course all those birds caught my attention, I had no idea what they were, and the little frogs singing at night by my window. But then they started filling in (the wetland)… And I was just a spectator… I said, all right! I promise I will be the voice of the species that live in the wetland. And that is where all of this begins…”* (Interviewee, with local leader Chimalfe wetland, Hualpén).

### 3.4 Socio-environmental transformations associated with the effects of climate change

Across all territories, the testimonies show that communities perceive the effects of climate change through the transformations they identify in the environmental cycles and conditions of everyday life, mainly through observation and direct lived experience in the territory. Communities identify this phenomenon through a marked variability and intensification of climatic events.

In rural and forestry territories, interviewees emphasize the presence of harsher winters with long periods of continuous rainfall and prolonged frosts that affect ecosystems, agricultural production, and daily life. Most interviewees also identify longer summers and a general increase in temperatures, which alter the traditional conditions of the regional climate and make it more vulnerable to other effects of global change.

*“They are winters, I mean they are more extreme, this past winter was really harsh, there were many straight days of rain and many straight days of frost, the frosts just would not stop… so that is when conditions are becoming more extreme”.* (Interview Buena Cabra leader, Santa Juana).

*“And I think the weather, the weather today is hotter, think about it, the climate has gone up because the heat is much stronger.”* (Interviewee, local leader La Aguada organization, Yumbel).

In the Andean foothill sectors, the testimonies point to a reduction in snowfall and the progressive disappearance of glaciers and snow reserves that used to persist for much of the year. These changes combine with other increasingly unpredictable phenomena, such as intense windstorms or rainfall occurring in seasons different from those that usually characterize these territories. (Interview enrivonmental activist, La Orilla, Quilaco).

In urban areas, the testimonies link the increase in temperature and greater heat stress to the transformation of the urban landscape, especially because of the loss of vegetation cover and the increased filling of wetlands driven by real estate expansion.

*“So, speaking about Concepción as a whole, I think you can definitely notice it… You can see that the temperature across the city has gone up. I think over the years climate change is being felt… also, there is less vegetation cover, because green areas such as the wetlands are increasingly being filled in to build apartment blocks. So you end up with fewer places that can mitigate the heat…”* (Interviewee, Paicaví leader wetland organization).

The geonarratives of climate change reveal a strongly territorialized understanding of this phenomenon in the study region, where communities not only identify its environmental effects and impacts, but also the tensions it generates in the socioecological resilience of ecosystems and human settlements, where climate change is intensified by other processes of global change such as real estate expansion, and in rural sectors by the presence of extensive forest monocultures that transform the natural and social conditions for responding to this new scenario. However, local populations are also recognized as having limited knowledge regarding climate change as a phenomenon and its impacts.

### 3.5 Territorial transformations derived from socio-environmental interventions, extractive dynamics, and processes of local overexploitation

In the territories where community climatic experiences are located, communities identify the expansion of large-scale exploitation projects and intensive land use change as one of their main socio-environmental problems, including hydroelectric and agroindustrial plants in the Andean foothills, wind farms in the drylands, and real estate, fishing, forestry, and oil infrastructure in coastal zones. However, across all testimonies, the predominant impact of forestry plantations stands out as the main concern, since their expansion and intensification in recent years constitute a persistent source of concern because of their territorial effects and the transformations they generate in the everyday life of communities:

*“…the forestry companies, as we know, have a system of desertification, they absorb underground water tables… every time we see river flow, let us say, in the seasons when water levels drop, I think that is squarely the fault of the forestry companies”.* (Interview Environmental activist, La Orilla, Quilaco).

The testimonies from all territories coincide in indicating that the main causes of the socio-environmental crisis of the region originate in these dynamics of overexploitation and extractivism, while climate change is regarded more as a phenomenon that amplifies and aggravates the environmental vulnerabilities previously produced by existing processes of environmental damage:

*“…I still do not see… a direct relation between climate change and the structural changes in the ecosystem, except maybe in some lagoon or some wetland that has dried up… But what has really been noticeable, more than climate change, is the transformation of the landscape generated by economic development and urban expansion”* (Interview Birdlife activist, Concepción).

In several of these territories, extractive projects are heterogeneous, and their impacts also generate synergies in the territories, which makes it difficult for communities to respond to all of them at once because of the magnitude of their impacts and the power relations through which they reconfigure everyday life in these territories (7).

*“…the forestry business, the real estate business, the ports have also generated changes, in Lirquén what the coast used to be was changed… the port of Talcahuano has also gone through changes… the issue of fishing industry extraction has also had an impact on marine resources”* (Interview Birdlife activist, Concepción).

In this context, some pluriversal experiences choose to focus on defending the territories from one type of company because of their limited capacities as community organizations:

*“But really, we are only fighting the company itself (the dam), because at one point we saw that we also wanted to get involved with the sawmill because of the chemicals. But what happens with the forestry company? What happens? You would have to fight two beasts at once, and it is better to fight one. Yes, because they are lethal, two lions are lethal, just one lion…” Interview* (Local leader organization defense of Laja river and environment, La Aguada, Yumbel).

A relevant finding in several experiences points to the difficulties involved in strengthening local environmental care practices among the local population of these territories. In most of the experiences studied, the proliferation of illegal dumping sites and the deterioration of ecologically valuable spaces through use by the local community or by visitors can be observed. This reflects gaps in awareness and environmental education at the local level, as well as structural deficits in waste management and in policies that support ecosystem care (Interview representative of Malen Leubu, Alto Biobio).

Other forms of local overexploitation also occur, although to a lesser extent, through indiscriminate gathering of fruits, mushrooms, and other foods traditionally collected in the local economies of rural sectors, as in the Andean foothills:

*“…everything related to gathering too, because while there is no… training in how to gather from the abundance of food we have around us, that has also caused certain mushrooms, for example, to disappear, like the morchela [morel]… And the same thing is happening with changles, and the same thing will happen too, hopefully it will not…”.* (Interview environmental activist, Rukamaya, Quilaco).

### 3.6 Climate–extractive entanglements: when both phenomena reinforce each other and complicate the environmental crisis

In some territories, specifically in sectors affected by flooding associated with climate change, synergies arise that intensify the occurrence of socio-natural disasters. These events not only generate direct impacts, but also amplify the accumulated effects of large-scale extractive activities already present in the territory. In this way, floods operate as catalysts that deepen environmental vulnerabilities, showing that they are inscribed within a broader climate–extractive entanglements:

*“…I think all of that goes hand in hand with climate change and with the issue of all the sediment that is building up (because of the dam), and over the years one thing supports the other”* (interview leader Local leader organization defense of Laja river and environment, La Aguada, Yumbel).

Without doubt, all of this demonstrates a strong observational capacity that forms part of the emotional and socio-environmental interrelation of local communities. In the dryland and Andean foothill territories, people identify the effects of climate change more clearly, whereas in coastal experiences there is greater questioning of whether this phenomenon should be considered the cause of the environmental crisis of the territory. More than climate change, the testimonies generally indicate that the central problem lies in the extractive projects that have affected these places. This is because those territories have been more strongly affected by urban expansion, the installation of large port, energy, and real estate projects, and by the existence of pine and eucalyptus monocultures around cities and towns:

*“…wow, it is hard to say this happened because of climate change, because most of the problems are due to direct human action. For example, there are species that are endangered like the red-tailed hawk, and the red-tailed hawk is a bird that lives in the forest and sometimes adapts to pine trees, but then the pine trees get cut down anyway… its population has mainly been reduced because of deforestation, you know, so climate change is not the problem”* (interview Birdlife activist, Concepción).

A very illustrative case is the appearance of waterspouts (tornadoes) in the Bay of Concepción in recent years, in a territory where these phenomena did not previously occur, but where there are also pre-existing impacts from forest monocultures. The expansion of extractivisms in these territories increases disaster risk because of their convergence with the effects of climate change already experienced by coastal communities, which has also placed their lives and homes at risk, as shown by the episodes of waterspouts in the Bay of Concepción:

*“…and climate change worries me too, along with the expansion of ENAP, because there was a project to expand ENAP and at that time they did not accept our observations. I was inside the wetland when the waterspout happened… and when it was coming, I said wow, I am going to run home… it came and did not make it as far as O Higgins Avenue, which is the entrance up there in the northern part… It went that way and then toward the mall, and it did not hit the wetland, it did not go in”* (Interview leader Chimalfe wetland, Hualpén).

Yet the complexity and confusion surrounding many of these phenomena, and the heterogeneity of factors involved in climate–extractive entanglements, mean that in some cases actors state that they truly do not know what to attribute the effects of the environmental crisis to, as for example with the increase in drought in Mulchén, where they simply do not know whether it is due to the climate crisis or to forestry plantations (Interview with organic farmer, Mulchén).

### 3.7 Community experiences and dynamics of socioecological resilience

The community experiences of socioecological resilience undertake self-managed initiatives oriented toward the socioecological and restorative care of place, as well as environmental education initiatives. These two groups of initiatives are not practiced as separate spheres, but instead represent part of an overall understanding of the ways of caring for the territory expressed by most of these experiences.

In the first group, these are practices that combine numerous restorative care actions oriented toward improving, regenerating, and protecting socioecological spaces of environmental and cultural value:

*“In fact, we have a native reforestation project with the board… many people have already planted, have already fenced areas… another of the ways we see the forest reproducing more quickly is by fencing it off…”* (interview representative Male Leubu, Alto Biobío).

As for environmental education activities, these cut across all pluriversal experiences, where the conditions of environmental degradation affecting the territories and their complex multicausality are made visible. The aim is to make these realities visible so that local communities and visitors become aware of these conditions and of the ecological value of the territory, as well as of the environmental damage it currently exhibits. In many cases, the local community is not familiar with the biodiversity spaces of the territory or their relevance:

*“…we asked that all neighborhood associations, all the leadership teams, be taken to visit, say, the Sanctuary (of nature). We organized many outings so they could see the cliffs, the number of birds they had never seen, and the natural beauty. We took them around our municipality… because there are many people who live here but do not know their territory…”* (Interview Birdlife activist, Concepción).

In some experiences, spaces of care can be identified that carry out restoration actions and design sustainable ways of inhabiting, which also function as demonstrative spaces for environmental education:

*“Permaculture is a design tool that we are using precisely to design this space, to zone this space. And at the same time, in terms of construction, we use one of the tools of permaculture, which is bioconstruction, and that supports all the sustainable construction we are doing, since we do not use materials that harm nature.”* (Interview environmental activist, Rukamaya, Quilaco).

There are experiences that produce organic foods such as vegetables and honey in Pehuenche communities and peasant farms:

“Here we produce potatoes, corn, broad beans, peas, lettuce, tomatoes, cabbage, cauliflower, beetroot, in the summer tomatoes… So I think that, and using good fertilization systems, because we have vermiculture, we prepare humus plus sheep manure, we make a mixture and with that we can produce good fertilizers, ensuring that what we put into the soil will not damage it and therefore will not damage us either… …” (Interview leader rural water association Biobio Province, Santa Bárbara).

This collective actionsintegrate care practices in both their immaterial dimensions—such as memories, meanings, and knowledges—and in concrete actions of socioecological restoration. These include community initiatives such as the cleaning of contaminated spaces, agroforestry, ecological agriculture, and seed exchange (*trafkintu*). In Pehuenche communities of the Andean foothills, these practices are grounded in Indigenous cosmovision and in the revaluation of ancestral knowledges, especially in seed care and more ecological food production:

“*We also try to hold trafkintu gatherings in the community in Cauñicú (care and exchange of seeds), where people talk about the importance of ancestral organic seed and also about environmental care and the problems we face… there is always an attempt to foster self-management and collective organization in favor of environmental care, the local economy, and the conservation of culture.”* (Interview representative Malen Leubu, Alto Biobío).

In some wetlands in coastal sectors, local organizations themselves have restricted problematic uses of certain places in order to protect them and thus halt the damage they are subject to, while still preserving community access:

*“…another part of the care work we do in the wetland is blocking vehicle access… we put in posts… we build a fence and paint it yellow so it can be seen from far away, so it does not stop pedestrian access if someone wants to go for a walk, but at least it stops the trucks and pickup trucks that go there to dump rubble…”* (Interview leader Paicaví wetland, Concepción)

### 3.8 Socioecological resilience in the face of the environmental crisis

The generative testimonies show that socioecological resilience is built through the interrelation and ecodependence between communities and the ecosystems they inhabit and care for. In these cases, the systematic observation of the environment carried out by communities emerges as a fundamental practice for understanding local ecological dynamics, and the interpretations derived from it guide socioecological actions of care, management, and adaptation. The everyday observation of territory makes it possible to identify emerging environmental changes in place, as well as to adjust productive practices and strengthen strategies for responding to socioecological disturbances.

*“And those changes have to be observed, recorded, looking at how the space behaves from one year to the next, how it also changes from one season to another, how, for example, the trees are leaning toward the sun, what different foods we can gather. So we have to make an everyday and constant reading of what the space is.* (interview of environmental activist Rukamaya, Quilaco).

The community experiences identify changes in the conditions of the territory, recognizing that “the climate is not what it used to be.” Through direct observation, they also adjust productive and care practices, for example by modifying work schedules or cycles of territorial care, and by adapting uses and practices. These decisions reflect adaptation processes based on experiential knowledge of territory that, in scenarios of crisis, become relevant as mechanisms of adaptation by reorganizing practices, in this case productive ones:

*“…You can tell that the climate is not like it used to be, and when working in the orchards themselves, I try to avoid, for example… the peak hours, when you know the sun is very strong…. I always work in the afternoons, avoiding the sun, avoiding the high temperatures. Well, in winter, in the greenhouse you can work… so you have to keep adapting to the climate… that adaptation process more closely associated with working the land…”* (*Interview, leader rural water association Biobio Province, Santa Bárbara*).

In these experiences socioecological resilience is a situated process that results from joint work between community and nature, that is, it is a coproduction between both. In this process, dialogues of knowledges play a key role in the construction of knowledge, articulating local knowledges, collective learning, and community self-training. These epistemic processes contribute to the generation of adaptive responses to the socioecological transformations of territory, reinforcing the capacity of communities to sustain practices of care and ecosystem management in contexts of change.

*“…I learned to move within that cycle, within the cycle of the seasons…. As you come to understand how the cycle of nature moves, you also adapt to flow with that, let us say, movement. Here, for example, in autumn I go out to collect mushrooms, and there is also the importance of studying which mushrooms you can collect, which you cannot, how they are prepared, how they can be preserved…”* (Interview environmental activist Catherine, La Orilla, Quilaco).

### 3.9 Future projections in the face of the environmental crisis from pluriversal territories

In general, there is a transversal concern about the future of these territories based on their current socioecological conditions, although there is no consensus regarding climatic and environmental projections. Pluriversal experiences express divergent perspectives, since some anticipate irreversible deterioration, while others project that the crisis will generate greater awareness and environmental care. There is also concern about the expansion of large-scale economic projects—such as forestry plantations—promoted by public policies in the region, which intensify socio-environmental threats and strain pluriversal visions of territory (Table 1).

**Table 1:** Synthesis of the category-based analysis.

Category of analysis	Main findings
Socio-environmental characteristics of the territories	Different levels of conservation, with biodiversity refuges at risk. Andean foothills: high conservation under threat (hydroelectric and forestry projects), pehuenche Indigenous territory. Drylands: water crisis, forestry and hydroelectric projects, fragmented forest. Peasant territories. Coast-littoral: peri-urban territories, biodiversity in estuaries, threat from urbanization and forestry plantations.
Socioecological interrelations, pluriversal communities, and territory	Territory as a material and symbolic support that articulates identities and affects. Diverse forms of rootedness (ancestral-Indigenous, historical, and migrant). Wetlands as key common goods. Rootedness in contexts of threat strengthens bonds and commitments of care.
Transformations associated with climate change	Higher summer temperatures and drought, colder winters. Changes in seasonal cycles. Increase in waterspouts. Climate change aggravates pre-existing environmental problems. It is not their main cause. Limited knowledge of the effects of climate change in the region.
Transformations derived from extractive dynamics and overexploitation.	Expansion and presence of large-scale and heterogeneous extractive projects. Forestry plantations are regarded as the main threat. The environmental crisis is caused by extractive dynamics, and climate change acts as an amplifying factor. Mutually synergistic impacts. Environmental defense is limited to certain threats because of restricted organizational capacities. Difficulties in strengthening socio-environmental care, with illegal dumping sites and contamination due to lack of environmental education and deficient local environmental management.
Community experiences and socioecological resilience	Environmental education, socioecological restoration, agroecology, and seed care. Local lack of knowledge about some biodiversity spaces and their importance. Forms of care that articulate memories and knowledges with concrete restoration actions. Restriction of harmful uses in ecologically sensitive places for their protection.
Socioecological resilience in the face of the environmental crisis	It results from the interrelation and coproduction of communities and ecosystems. Resilience based on local observation, collective learning, and the adaptation of practices to environmental change. Adjustment of productive and care practices through the reorganization of productive uses. Dialogue of local knowledges, collective learning, and community self-training. Self-managed initiatives for the socioecological and restorative care of place.
Future projections of the territories	Divergent perspectives, with some experiences anticipating irreversible deterioration. Other experiences project the future as a space of hope and greater awareness. Environmental education is indispensable for creating awareness. The future depends on it.

*“From the moment this ecological awareness is not generated, not only will more power plants arrive, forestry companies will arrive, wind farms will arrive, photovoltaic panels will arrive, geothermal mining explorations will arrive, and there are already more than four hundred and thirty-two of them here in the province”.* (Interview representative Isla Nuestra, Santa Bárbara).

One group of testimonies presents the future as a space of hope where better scenarios are expected to emerge, and some actors state that this is a slow process but that it is happening, as awareness of the relevance of environmental care has been increasing, particularly through a change in the way humanity thinks, especially among younger generations (Veronica, agroecología Mulchén). From this perspective, recovering the ecosystem functions of spaces of care such as wetlands is considered crucial, as this is a key aspect of the environmental restoration of these places:

*“…I have hope because restoring the ecosystem is key to confronting this change, this triple climate crisis… Restoring the parts of a wetland that have been damaged so it can function. The same in the hills, with native forest and native vegetation, to prevent erosion.”* (Interview leader Chimalfe wetland, Hualpén).

Hope is not an isolated aspect. The geonarrative accounts consider it viable through the strengthening of environmental education in order to deepen community awareness, and in that way to promote the creation of spaces of organization, sustainable planning for the use and restoration of territories, and the search for limits capable of slowing the environmental crisis:

*“The only path is education. And to achieve that, the main thing is to create spaces… the real purpose is for people and society to take over spaces and organize… and begin to realize that we do not have much time left… that they truly feel the need to create change.”* (Inteview environmental activist, La Orilla, Quilici).

## 4 Discussion

The results show that the pluriversal experiences analyzed cannot be understood through conventional frameworks of climate vulnerability or resilience, but rather through their socioecological configurations, their community–nature relational dynamics, and their historical territorial trajectories. In this sense, the Biobío region constitutes an emblematic case because of its history of dispossession and conflict, as well as its conditions of social activation, structural precarization, overexploitation, and environmental degradation, all of which favor the emergence of multiple climatic pluriverses.

Recognizing the contextual differences of each territory, the analysis identifies fewer pluriversal experiences in the dryland valleys of the region and a greater concentration of these experiences in the coastal-littoral zone of Greater Concepción. This is related to the emblematic socio-environmental conflicts in these territories, the greater density of socio-environmental critical mass—both because of hydroelectric dams in the Andean foothills and because of heterogeneous forestry and energy conflicts along the coast-littoral—as well as the historical processes of dispossession and accumulated resistance in these places, among other factors.

In the experiences of community socioecological resilience, socioecological restoration, environmental education, and sustainable practices of food production and ways of inhabiting, such as agroecology and permaculture, predominate. These practices represent a field not only of actions, but also of relations, knowledges, affects, and memories that are activated in response to the environmental crisis in each territory.

Regarding climatic pluriverses, this concept represents a decolonial framework for understanding community alternatives to the environmental and climate crisis from the historical trajectories of their territories, their community–nature relational dimensions, their ontological dimensions, and their socioecological configurations, thereby moving beyond traditional climate approaches. These experiences demonstrate transformative potential at the local scale, although they face difficulties in moving beyond place, yet they still hold the potential to scale into broader frameworks of influence. They are experiences that effectively emerge from interdependence with diverse human and nonhuman forms of life (12), and they are highly situated and dependent on the diverse contexts of environmental threats and local cultures of each territory, expressed through practices of care, rootedness, defense, and creation that articulate socioecological resilience through such actions.

Pluriverses are heterogeneous and are not free of conflict, since divergent visions, discourses, and strategies coexist within them, particularly regarding the future of their territories, although they share the recognition of the need to broaden social awareness of the crisis and to strengthen environmental education in order to enhance socioecological resilience. Diverse forms of relationality can be observed according to the conditions of the Andean foothills, the coast, and the drylands, in which nature takes different forms that persist through the possibilities of resilience enabled by the climate-extractive assemblages that predominate in each place.

Pluriversal actors construct diverse routes of relationality (25) with territory—local actors native to the place and outsiders who have settled there—and with their ways of signifying nature. In scenarios of such severe crisis and such complex and dynamic climate-extractive assemblages, pluriverses are configured as spaces of negotiation, conflict, and coexistence among diverse territorial ontologies. Many of these are marked by the experience of building identity-based attachment to place, both among those native to the territory and among those who migrated to it.

Regarding climate-extractive assemblages, the findings show that they constitute a key analytical category for understanding the complexity of the territorial scenarios from which climate pluriverses emerge, since they make it possible to account for the synergistic interaction among the effects of extractive economic activities, the effects of climate change, and some local forms of overexploitation, whose effects have accumulated historically in each place (53).

In the pluriversal experiences of Biobío, the environmental crisis is conceived as a complex and multifactorial whole in which the different causal elements are not understood in isolation, but as an interrelated totality, although with clearly differentiated responsibilities. In this context, and within the climate-extractive assemblage, the testimonies coincide in recognizing the expansion of forestry plantations as one of the main drivers of socio-environmental degradation in the region, especially in rural territories. Meanwhile, in the urban and peri-urban sectors of the coast, responsibility falls more heavily on real estate expansion, although the diversity of problems that converge in each place is always acknowledged.

As a territory characterized by historical processes of dispossession and high environmental degradation, the Biobío region does not understand climate change as the main cause of the crisis, but rather as a factor that intensifies and deepens pre-existing territorial transformations (53). Nevertheless, the rise of climate discourse and of public policies aimed at adaptation to this phenomenon has opened opportunities to promote adaptation, prevention, and restoration actions, as well as to make socio-environmental problems of anthropogenic origin more visible. It should be noted that important gaps in public information and awareness regarding these problems persist, together with a certain caution and distrust among pluriversal experiences regarding the possible instrumentalization of climate change as a mechanism for obscuring the structural role of extractivism in the environmental crisis of the study territories.

In this context, climatic pluriverses and forms of socioecological resilience emerge from the crisis of lived experience amid these assemblages, which stimulate the construction of relational and situated responses that articulate different dimensions of the crisis through the experience of caring for place. However, the high complexity and multicausality of the environmental crisis sometimes tend to fragment community experiences, forcing them to focus on specific problems because of their limited capacities to address so many simultaneous threats, and because of an overload of actions that in many cases exceeds their capacities for intervention.

Regarding socioecological resilience, the pluriversal experiences of Biobío show that this idea requires a broader reinterpretation as a relational practice of coproduction between communities and nature for the care of territory, rather than as an exclusively human action. The experiences studied show that socioecological resilience emerges from the dialogue between ecosystems and human experiences, mediated by biocultural attachments involving affects, identities, territorial memories, and the generation of situated knowledge from direct experience with place. In this way, resilience is configured through relations of inter-ecodependence (58) and reciprocity between actors and their environment.

In this sense, resilience is neither exclusively community-based nor exclusively biophysical, but rather a socioecological coproduction that takes shape in contexts traversed by highly complex and dynamic climate-extractive assemblages, where communities coproduce multiple forms of agency with nature. The lived experience of territory, the forms of relationality and emotionality in the Andean foothills, drylands, and coast, as well as the social fabrics and local knowledges, are fundamental for the configuration of resilience.

## 5 Conclusion

This research shows that the Biobío region represents a particularly fertile scenario for the emergence of climate pluriverses, insofar as a high intensity of socio-environmental conflict converges there with a sustained trajectory of ecosocial activisms. It is a space historically shaped by territorial disputes and multiple extractive projects—especially forestry, energy, and industrial projects—whose effects intertwine with the recent impacts of the climate crisis, thereby configuring conditions of high socioecological complexity. In this context, creative community responses are activated and expressed through forms of organization, care, and ecological restoration based on relational bonds with nature.

Across the territory, we find a high production of pluriversal experiences in coastal and Andean foothill territories, and to a lesser extent in the inner dryland valleys, which responds to the heterogeneity of their historical, cultural, and ecological processes. The pluriversal experiences of the region configure active forms of socioecological resilience, anchored in deep territorial rootedness and in relations of inter-ecodependence with the ecosystems of each place. These initiatives are oriented mainly toward socioecological restoration, environmental education, and agroecological production as their principal axes of action.

Pluriversal strategies not only confront the effects of climate change, but above all confront the accumulated impacts of multiple extractive activities present in the territories. From these experiences, socioecological resilience is configured as a dynamic process based on the mobilization of local knowledges, collective learning, and situated practices, stimulated by relations of lived affect with local ecosystems and by the urgency of the effects of the environmental crisis. That is, it is a coproduction between community and nature.

A central finding is the identification of climate-extractive assemblages as platforms of action where the climate crisis intertwines with the historical dynamics of environmental degradation and dispossession, which tend to deepen the environmental and climate vulnerability of communities. Despite this multicausality of factors, communities recognize that the effects of climate change intensify the impacts of environmental degradation that are produced fundamentally by extractive economic activities, rather than by the environmental crisis as a principal cause.

Although these experiences face limitations associated with the precarious conditions in which organizational action takes shape, dependence on local leadership, limited institutions of environmental protection, and contexts of high social vulnerability, climate pluriverses open alternative horizons for enriching public discussion oriented toward an interpretation of the local scenarios in which the effects of climate change are expressed, as well as for constructing responses for local climate action from situated and socio-natural approaches. They also contribute to decentering strongly technocratic visions of resilience and climate action, proposing an expanded repertoire of adaptation responses that includes pluriversal forms of care based on inter-ecodependence, biocultural territorial knowledge, and the diversity of practices through which communities cocreate with nature.

## Data Availability

The original contributions presented in the study are included in the article/Supplementary material, further inquiries can be directed to the corresponding author.
