# Supplementary material for: Climatic pluriverses: community contributions to socio-ecological resilience from intercultural territories within climate-extractive entanglements in south-central Chile
Source: Front Public Health. 2026 Jul 30;14:1841434. doi: 10.3389/fpubh.2026.1841434 (PMC13469249; doi:10.3389/fpubh.2026.1841434)
Supplement: Supplementary file 1 [file Supplementary_file_1.DOCX]

**APPENDIX**

**Table of pluriversal experiences Biobío region, Chile interviewed and visited**

| **n°** | **Group or community** | **Municipality, town** | **Experience descriptions** |
| --- | --- | --- | --- |
| 1 | Defensa Ambiental La Orilla | Quilaco,/ rural/precordillera | Agroecological gardens, Regeneration earth, environmental activism, environmental education. |
| 2 | Comité Isla Nuestra, Santa Bárbara | Santa Bárbara / rural/ precordillera | Environmental activism, restoration native woods, wetland care, environmenal education |
| 3 | Organización socioambiental Malen Leubu | Alto Biobío / rural/precordillera | River’s care, environmental education, environmenal activism, cultural development pehuenche towns, womens rafting. |
| 4 | Centro Demostrativo de Vida Sustentable: Permacultura y Bioconstrucción Rukamaya | Quilaco / rural / precordillera | . ecological conservation, environmental education, permaculture, environmental activism. |
| 5 | Centro de Educación Ambiental y terapéutico Cantos del Universo Ulkantún Wenu Mapu | Alto Biobío / rural / precordillera | Apiteraphy, ancestral knowledge indigenous Mapuche-Pehuenche with holistic medicine.  Woods native care, environmental activism. |
| 6 | Agroecología Mulchén | Mulchén / rural / secano-precordillera | seed care with this family, grow vegetals, regenerative soil improvement. Environmental and agroecological education. |
| 7 | Asociación de APR Provincia del Biobío | Los Ángeles, provincia del Biobío./rural / secano-precordillera  . | Rural water association province of Biobío, Asociaciónwater rural care,  environmental education of water. . |
| 8 | Comité de Defensa Humedal Paicaví | Concepción / semiurban / coast | wetland activism, environmental education, restoration, guided tours, Actividades de defensa del humedal. |
| 9 | Birdwatching Biobío | Concepción / semiurban / coast | Environmental education, environmental activism, birdlife activism. |
| 10 | Comité de Defensa y Restauración humedal Vasco de Gama - Chimalfe | Hualpén/ semiurban / coast | activism wetland, environmental education, , wetland care. |
| 11 | Buena Cabra | Santa Juana,  Chacay / rural / secano | Agroforestry, ecological prevention of wildfire, environmental education, design resilient landscapes, ecological grazing |
| 12 | Agrupación por la defensa del río Laja y el medio Ambiente | Yumbel / ural / secano | environmental activism, restoration, cultural activities, care of Laja river. |
